# Placental Blood‐Flow Velocity Quantification From Diffusion MRI

**DOI:** 10.1002/mrm.70551

**Published:** 2026-08-10

**Authors:** ZhuangJian Yang, Diana Cruz De Oliveira, Leevi Kerkelä, Marco Palombo, Elizabeth Powell, Christopher S. Parker, Daniel Cromb, Lisa Story, Serena J. Counsell, Kelly Payette, Joseph V. Hajnal, Jana Hutter, Rebecca J. Shipley, Daniel C. Alexander, Paddy J. Slator

**Affiliations:** ^1^ Hawkes Institute and Department of Computer Science University College London London UK; ^2^ Department of Mechanical Engineering University College London London UK; ^3^ Cardiff University Brain Research Imaging Centre (CUBRIC), School of Psychology, Cardiff University Cardiff UK; ^4^ School of Computer Science and Informatics Cardiff University Cardiff UK; ^5^ Hawkes Institute and Department of Medical Physics and Biomedical Engineering University College London London UK; ^6^ Early Life Imaging Department School of Biomedical Engineering and Imaging Sciences, King's College London London UK; ^7^ Department of Women and Children's Health School of Life Course Sciences, King's College London London UK; ^8^ Biomedical Engineering Department School of Biomedical Engineering and Imaging Sciences, King's College London London UK; ^9^ Center for MR‐Research University Children's Hospital Zurich, University of Zurich Zurich Switzerland; ^10^ Institute for Information Processing Leibniz University Hannover Hannover Germany

## Abstract

**Purpose:**

Altered placental capillary blood flow is closely linked to obstetric complications, yet quantifying capillary‐scale blood velocity remains challenging with existing imaging methods. This is partially because capillary networks form disordered microvascular beds at the voxel scale, rather than coherent, directional vessels. Here, we combine Monte Carlo (MC) simulations with machine learning to estimate placental capillary blood velocity directly from diffusion MRI (dMRI) data.

**Methods:**

MC simulations incorporating perfusion and diffusion were performed to generate signal dictionaries for supervised machine learning regressors (random forest [RF] and multilayer perceptron [MLP]) to estimate velocity, perfusion fraction and diffusivity. The trained regressors were applied to simulated and in vivo dMRI data, with intravoxel incoherent motion (IVIM)‐based estimates as baselines for comparison.

**Results:**

Our approach outperformed IVIM‐based methods on simulated test data in recovering ground‐truth parameters. Among the evaluated models, the MC‐based MLP approach produced physiologically reasonable estimates of velocity in in vivo placentas, in agreement with previously reported measures and exhibited greater sensitivity than IVIM‐generated methods.

**Conclusion:**

This study presents an approach to characterize capillary perfusion that alternates conventional IVIM models, particularly in organs such as the placenta, where pseudo‐diffusion coefficient can be similar to tissue diffusion coefficient, limiting reliable IVIM parameter separation. Our method could enable detection of capillary blood‐flow alterations, which could help identify pathological placental blood‐flow conditions and potentially diagnosis relevant diseases, such as preeclampsia and fetal growth restriction. More broadly, the approach may translate to other organs where microscale blood‐flow changes are key indicators of underlying pathologies.

## 1 Introduction

The placenta plays a central role in the long‐term health of both fetus and mother [1]. Placental blood flow is crucial for fetal development, and disruptions in placental perfusion are linked to major obstetric complications, including preeclampsia and fetal growth restriction (FGR) [2]. Despite the importance of monitoring and understanding placental circulation, the patterns of blood flow within its complex vascular architecture remain poorly understood. This is particularly pertinent at the microvascular level, due in part to the small size, dense interconnectivity, and heterogeneous orientations of capillaries, making direct measurement and imaging challenging compared to larger vessels [3]. Addressing this gap is essential for advancing diagnostic capabilities and improving maternal‐fetal health [4].

Non‐invasive clinical methods such as umbilical artery Doppler ultrasound [5] and uterine artery velocimetry [6] are commonly used to indirectly assess uteroplacental and fetoplacental hemodynamics, but they primarily reflect flow in large upstream vessels and lack the spatial resolution to detect flow at the capillary level (typically < 30 μm in diameter), where the exchange between maternal and fetal circulations occurs [7]. MRI‐based phase contrast angiography can measure blood velocity at the voxel scale (typically 1–3 mm^3^) and has provided novel insights into placental circulation, but is designed for coherent (directional) flows and requires bespoke sequences [8]. These limitations make phase contrast angiography unsuitable for quantifying the randomly oriented, incoherent (non‐directional) blood flow present in placental capillary beds. Furthermore, while some advanced imaging methods attempt to separate maternal and fetal blood flows [9] or incorporate T2* information [10] to improve placental assessment, they fall short of directly capturing complex and incoherent flow at the capillary level.

The IVIM model for diffusion MRI (dMRI) can capture perfusion in incoherently orientated capillaries [11]. However, IVIM models average incoherent flows across the entire voxel into a single scalar value, the pseudo‐diffusion coefficient (D*), which reflects the combined contribution of blood velocity and microvascular segment length (i.e., the Einstein relation: D* = L v/6), assuming that microvascular perfusion behaves as pseudo‐diffusion [12]. While this parameter can be used as an indirect marker of perfusion, this formulation limits the ability of IVIM to disentangle capillary flow hemodynamic properties of the placental microvasculature [13].

Recently emerging microstructure fingerprinting techniques combine Monte Carlo (MC) simulations with machine learning (ML) to extract more detailed tissue parameters than traditional models, such as neurite orientation dispersion and density imaging (NODDI) [14] or vascular, extracellular, and restricted diffusion for cytometry in tumors (VERDICT) [15], can provide. Microstructure fingerprinting generates extensive libraries of synthetic dMRI signals by simulating spin displacements in realistic tissue microstructure [16] and/or microvasculature [17, 18, 19] environments, which are then used to train machine learning algorithms to recover simulation ground truth tissue parameters. This combination of MC simulations and data‐driven inference enables a more detailed understanding of microstructure and microvasculature, capturing subtle variations that traditional averaging models overlook, potentially bridging the gap between current imaging capabilities and the need for detailed microvascular characterization in clinical practice [20].

Here, we apply the microstructure fingerprinting approach to estimate capillary blood velocity directly from in vivo dMRI of the human placenta. We construct simplified capillary geometries that retain key features governing dMRI signal formation and capture capillary‐scale flow effects relevant to diffusion attenuation. In parallel, we generate dMRI signals using the IVIM model, deriving effective pseudo‐diffusion coefficients from biologically relevant capillary flow velocities via the Einstein relation. These synthetic signals are used to train machine learning models (random forest [RF] and multilayer perceptron [MLP]) to estimate capillary flow velocity, perfusion fraction, and diffusion coefficient. Crucially, unlike traditional IVIM studies that aim to separate the overall intravascular and extravascular signals (and hence likely conflate maternal intervillous space, larger vessels, and fetal capillaries in placental applications), our framework focuses specifically on isolating the signal originating from the feto‐placenta capillary beds.

## 2 Methods

Section 2.1 introduces the in silico structures used to mimic incoherently‐orientated capillary structures. Section 2.2 describes a modified MC simulator that generates dMRI signals based on structures in Section 2.1 and how the IVIM model is used to generate similar datasets. Section 2.3 explains how MC‐simulated and IVIM‐simulated data are used to train machine learning algorithms to estimate the parameters of interest. Section 2.4 presents the in vivo data used in this study.

### 2.1 In Silico Structures

To capture key intravoxel flow behaviors within dMRI voxels, two different capillary structures were used in this study. The first is a 3D structure, which we denote as the “ring‐ball” structure, composed of three non‐intersecting rings, each formed from eight short cylindrical segments. This structure induces spin reorientations during a typical diffusion time along three coordinate directions, mimicking the directional changes occurring in curved or branching capillaries and effectively approximating the capillary structures assumed in the IVIM model. The second structure consists of randomly orientated cylinders in 3D implemented using a modified framework [21], producing an effectively isotropic distribution of capillary orientations. Although cylinders are not explicitly connected, this geometry captures incoherent flow‐related signal attenuation. This structure is akin to the VERDICT model [22], which represents vasculature as isotropically distributed “sticks” (cylinders with zero radius, while the randomly orientated cylinder structure have finite radius) to approximate in vivo microvascular behavior. Although not designed to directly replicate placental microvasculature, together these simplified geometries capture aspects of capillary‐scale flow that are known to influence dMRI signal generation in the placenta [12, 13]. Figure 1a shows a schematic of placental structure and Figure 1b displays the feto‐placental capillary structure our simulation is designed to mimic. Figure 1c is an example of the ring‐ball structure, where each ring comprises eight cylinder sections with selected lengths (L) and diameters (d) from biologically relevant ranges for placenta capillaries. Figure 1d illustrates an example of the randomly orientated cylinder structure, where cylinders with selected diameters (d) from the same range are randomly placed into the given cubic space. We tested whether inclusion of both geometries is necessary and whether each contributes to the diversity of the training dataset with a principal component analysis (PCA) (Figure S1). Although these structures are intended to capture capillary flow, they may also detect small‐scale maternal blood flow within the intervillous space.

**FIGURE 1:**
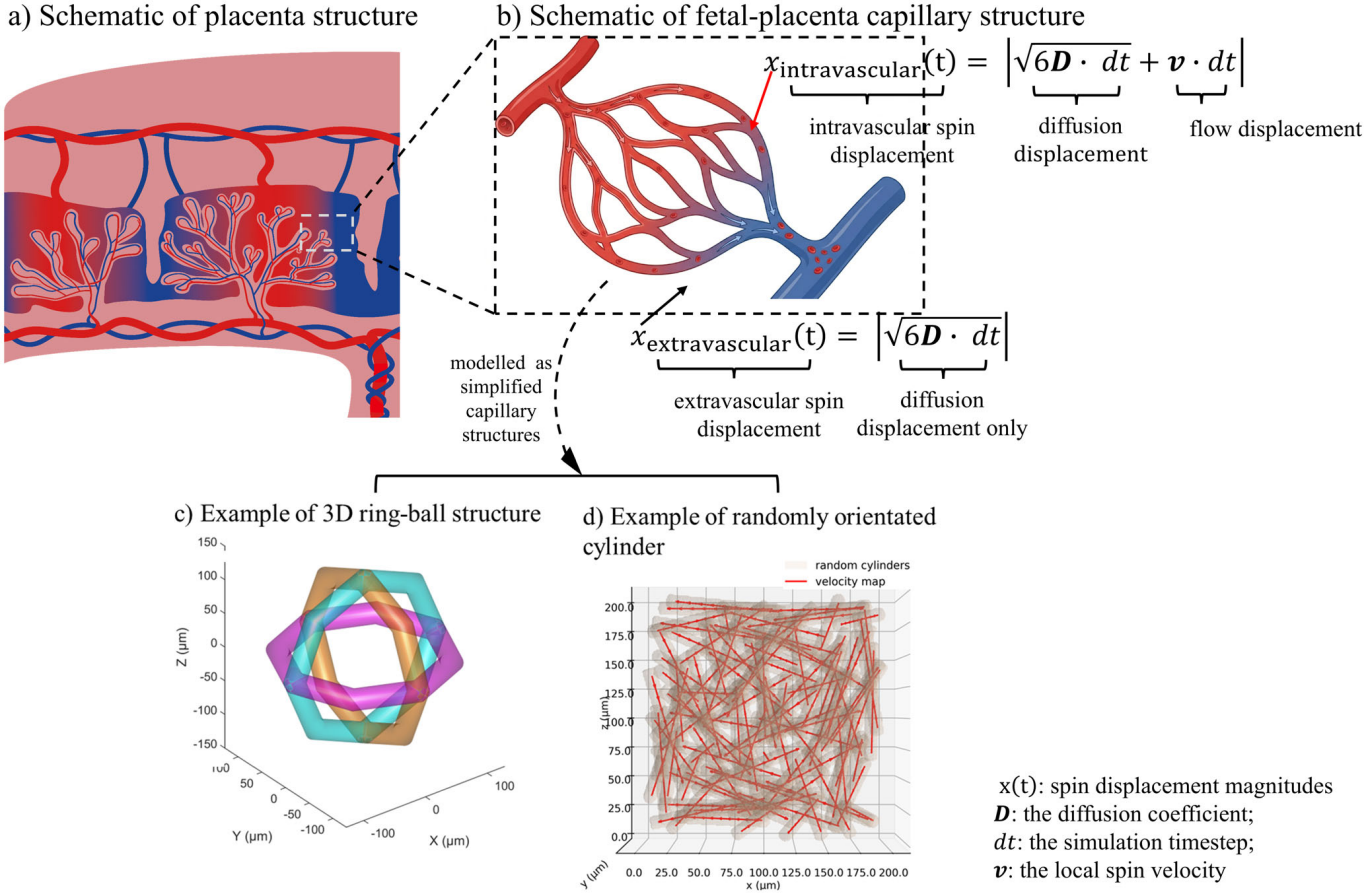
Schematic representations of (a) the placental structure and (b) the feto‐placental capillary structure. (c) Example of a ring‐ball structure, the velocity field follows an octagonal path causing spins to circulate within individual rings, each ring is isolated with no intersections, and (d) Example of randomly orientated cylinder structure, cylinders are shown with fixed ends but are effectively infinitely long in the simulations.

### 2.2 Simulation

#### 2.2.1 MC Simulation

We modified the open‐source Disimpy MC simulator [23] to simulate dMRI signals accounting for flow and diffusion within the defined capillary structures. For every Monte Carle perfusion simulation, within the ring‐ball or randomly orientated cylinders, spins move according to a velocity field that is aligned with the orientation of the cylinder section in which the spin resides with an additional random displacement governed by the diffusivity. On the other hand, outside the ring‐ball or randomly orientated cylinders, the non‐flowing component is modeled as unrestricted (free) water diffusion randomly based on a specified diffusion coefficient within the biological range. The boundaries of the structures are treated as impermeable surfaces with elastic reflection boundary conditions. Intervascular ($S_{i}$) and extravascular ($S_{e}$) spin signals were separately simulated within and outside both structures, and the overall signal ($S_{\text{sythetic}}$) was determined based on the perfusion volume fraction ($V_{f}^{\mathrm{MC}}$)

(1) $$S_{\text{sythetic}}=V_{f}^{\mathrm{MC}}\cdot S_{i}+\left(1-V_{f}^{\mathrm{MC}}\right)\cdot S_{e}$$

To avoid confusion with the IVIM perfusion fraction ($V_{f}$) we refer to $V_{f}^{\mathrm{MC}}$ as the MC perfusion fraction throughout. To simulate placental perfusion and diffusion, spin displacement magnitudes (*x*(*t*)) were modeled by incorporating both flow‐induced spin motion and diffusion‐driven random motion [23, 24]:

(2) $$x\left(t\right)=\left|\sqrt{6D\cdot \mathrm{dt}}+v\cdot \mathrm{dt}\right|$$

where D is the diffusion coefficient, dt is the simulation timestep, and v represents the local spin velocity. For extravascular signal estimation, only the diffusion term was considered (i.e., v = 0).

For signals generated using the 3D ring‐ball structure, parameters defining the ring‐ball were chosen within biologically relevant ranges for capillaries [25, 26]: cylinder length L = [10, 80] μm, cylinder diameter d = [5, 30] μm, velocity v = [0.001, 1] mm/s, and diffusion coefficient D = [0.001, 0.003] mm^2^/s. Although values outside these ranges may be possible, such as higher velocities in large vessels, the range used here represents a biologically relevant distribution for placental terminal capillaries. These precisely defined simulation input parameters serve as the ground truth of the simulated signals for training and evaluating the models in the subsequent sections. A total of 1000 parameter combinations were selected using Latin Hypercube Sampling with convergence checking, to reduce variance versus random sampling for small sample size [27].

When using randomly orientated cylinder structures, the signal generation used a fixed effectively infinite cylinder length, with spins initialized within the interior region (middle quarter) of each, and the simulation diffusion time and maximum blood velocities are insufficient for spins to reach the cylinder ends. The other parameters (d, v, D) were evenly distributed with 10 sample values within the same ranges as above, resulting in 1000 parameter combinations.

For both the 3D ring‐ball and randomly orientated cylinder structures, we ran a MC simulation for each of the 1000 parameter combinations, using a dMRI acquisition sequence from a previous study optimized for placental imaging [28]. The timestep used in the MC simulation was 2.6 × 10^−5^ s. We simulated 5 × 10^5^ spins per structure inside and 1 × 10^6^ per structure outside with convergence checking. For an NVIDIA GeForce RTX 4070 GPU, the 1000 ring‐ball simulations required approximately 11 days and 20 h to complete and randomly orientated cylinder simulations were more computationally expensive and required about 17 days and 18 h.

To synthesize the overall signals using linear combinations of the diffusion ($S_{e}$) and perfusion ($S_{i}$—from either ring‐ball or randomly orientated cylinder structures) signals via Equation (1), 10 volume fraction ($V_{f}^{\mathrm{MC}}$) samples were evenly distributed from 0 to 0.9 based on perfusion fraction values previously reported in the literature [29], resulting in 20 000 signals in the training dataset. Rician noise [30] was added to the final signals with signal‐to‐noise‐ ratio (SNR) equal to 20, matching a previous placenta dMRI study [31].

We synthesized a comprehensive test dataset covering the same range of simulation parameters described above. We also synthesized a secondary test dataset with extreme out‐of‐distribution parameter values, by extending the maximum velocity to 10 mm/s (we kept the same diffusivity and perfusion fraction ranges for this out‐of‐distribution dataset, since they already cover the whole physically reasonable range). We focus on evaluating prediction performance for capillary blood velocity, perfusion fraction, and diffusion coefficient, as our previous analysis showed that capillary length and diameter have a relatively minor influence on the signal within the biologically reasonable ranges mentioned above [32].

#### 2.2.2 IVIM dMRI Signal Generation

We also generated synthetic data using the IVIM model to assess how well an IVIM‐based approach can predict velocity, volume fraction and diffusion coefficient when their ground truth values are known. Although IVIM does not explicitly include velocity, we assumed the Einstein equation (D* = L v/6) to link capillary length and flow velocity to the pseudo‐diffusion [33]. As shown in Table 1, we used ground truth parameters with the same ranges of $V_{f}$, L, d, v and D as in Section 2.2. Each parameter was uniformly sampled at 10 points across its respective range, giving 10 000 parameter combinations. Thus, dMRI signals were generated by calculating D* from L and v using the Einstein equation and incorporating it with $V_{f}$ and D into the IVIM model equation ($S=S_{0}\left[\left(1-V_{f}\right)e^{\left(-\mathrm{bD}\right)}+V_{f}e^{\left(-bD^{*}\right)}\right]$). We simulated data via this IVIM model using the same dMRI acquisition weightings as the MC data except excluding *b* values > 1000 $s/{\mathrm{mm}}^{2}$ [11]. This is currently the best approach for estimating velocity directly from dMRI and shows correlations with μCT measurement of a vascular corrosion cast, although even μCT cannot fully resolve individual capillaries [33].

**TABLE 1:** Ground truth parameter ranges of Monte Carlo and IVIM simulation.

Parameters	L (μm)	d (μm)	D (mm^2^/s)	$V_{f}^{\mathrm{MC}}$/$V_{f}$ (−)	v (mm/s)	D* = L v/6
Monte Carlo simulation	[10, 80]	[5, 30]	[0.001, 0.003]	[0, 0.9]	[0.001, 1]	[1.7e‐6, 0.013]
IVIM simulation	[10, 80]	[5, 30]	[0.001, 0.003]	[0, 0.9]	[0.001, 1]	[1.7e‐6, 0.013]

*Note:* Randomly oriented cylinder structures used a fixed effectively infinite cylinder length preventing spin‐trapping at ends.

### 2.3 Parameter Estimation

To test if the MC simulation approach offers added value over the IVIM‐generated approach, we trained four distinct regressors by combining two regressors, RF and MLP, implemented in scikit‐learn, with both the MC simulation‐generated (described in Section 2.2.1) and IVIM‐generated (in Section 2.2.2) data. We denote these approaches MC‐based MLP, MC‐based RF, IVIM‐based MLP and IVIM‐based RF respectively. The RF regressor serves as a performance baseline, allowing us to evaluate whether the more complex MLP provides a measurable advantage across these geometries [34]. Hyperparameters of the RF regressor were optimized using GridSearchCV with RMSE between predicted and ground‐truth parameters as the scoring metric, employing 20% of the training dataset for cross‐validation. The optimal values were max‐depth = 40 and n_estimators = 1000 for MC simulation data, and max‐depth = 8 and n_estimators = 200 for IVIM‐generated data. Similarly, the MLP hyperparameters were tuned using the same procedure, resulting in a 4‐layer architecture (64, 32, 16, 5) for MC simulation data and a 3‐layer architecture (32, 16, 4) for IVIM‐generated data. For both models, the optimal learning rate after tuning was 0.005. The trained regressors were applied to the simulated test dataset described in Section 2.2.

### 2.4 In Vivo Data

The trained regressors were then applied to estimate parameters from in vivo placental dMRI data. The in vivo data, consisting of the first echo time from previously published diffusion‐relaxation MRI acquisitions [35, 36], employed the same diffusion weightings [28] as those used in our simulations (see Section 2.2). The protocol has *b* = [5, 10, 25, 50, 100, 200, 400, 600, 1200, 1600] s/mm^2^ shells with three gradient directions, a *b* = 18 s/mm^2^ shell with 8 directions, a b = 36 s/mm^2^ shell with 7 directions, and a *b* = 800 s/mm^2^ shell with 15 directions. This dataset included 78 scans from all healthy participants with manually defined ROIs encompassing both the placenta and basal plate and its gestation age were from 15.7 to 38.6 weeks. Participants were recruited in two ethically approved studies (Placenta Imaging Project [PIP], 16/LO/1573; and Congenital Heart Disease Imaging Programme [CHIP], 21/WA/0075), see reference [35] for full ethics details, inclusion/exclusion criteria, and how these were defined as controls. The trained model was applied voxel by voxel to each scan to generate parameter maps. A two‐stage fitting algorithm via least square IVIM fitting [37] implemented in Python was also applied to this in vivo data to check trends of perfusion fraction and diffusion coefficient during gestation of in vivo data and compared with them with our approach. The fitting assumed Gaussian‐distributed noise. The initial guess of D and D* were estimated from *b* values 200 to 600 and 0 to 40 (s/mm^2^) using monoexponential fitting [38].

## 3 Results

### 3.1 Evaluation of Machine Learning Regressors on Simulation Data

This section presents the performance of four models (MC‐based MLP, MC‐based RF, IVIM‐based MLP, and IVIM‐based RF) without and with noise (SNR = 20). For all comparisons, ground truth refers to the biological input parameters (mentioned in Section 2.2.1) used in the MC simulation or IVIM model. IVIM‐generated data are used as a baseline for comparative evaluation. As described in Section 2.2, model bias was assessed using a comprehensive test dataset covering all parameter ranges and structures, together with a secondary dataset extending beyond the nominal capillary velocity range.

Figure 2 shows 2D maps of estimation bias for velocity, perfusion fraction, diffusion coefficient across uniform parameter combinations (velocity vs. perfusion fraction, diffusion coefficient vs. velocity, and diffusion coefficient vs. perfusion fraction) of all four models (MC‐based MLP, MC‐based RF, IVIM‐based MLP, IVIM‐based RF). Across all mappings, the MC‐based MLP model provides the most accurate estimates (also see RMSE results in Table 2). MC‐based RF is the second best but fails in the diffusion coefficient—perfusion fraction map, while both IVIM‐based approaches perform poorly across all the plots. Similar patterns are observed when noise (e.g., SNR = 20) is added (Figure S2) and for the randomly oriented cylinder geometry (Figure S3).

**FIGURE 2:**
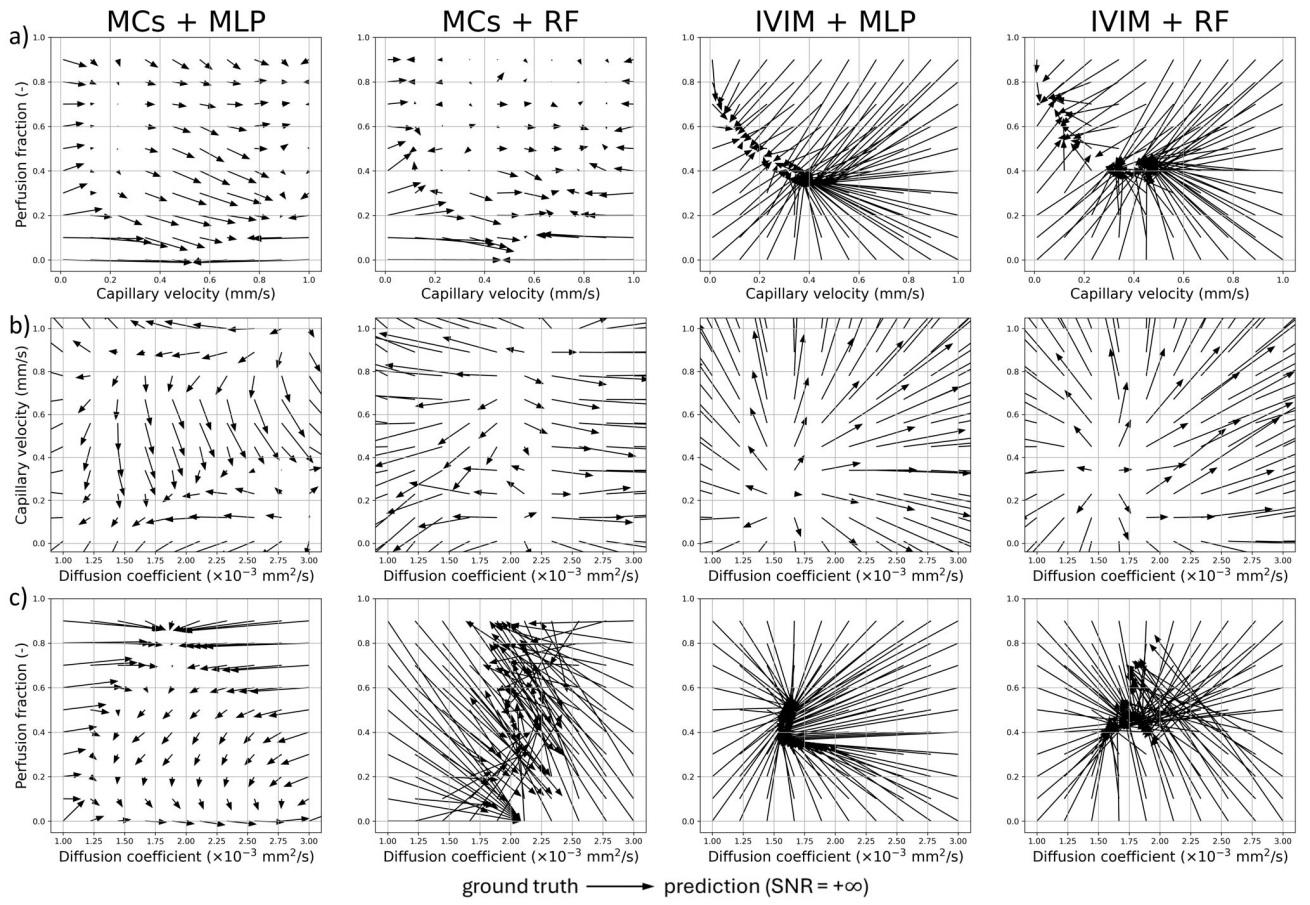
Prediction bias maps based on Monte Carlo simulation‐generated and IVIM‐generated RF and MLP regressors. Arrow tails indicate ground truth parameter values, while arrowheads indicate predicted values. For all plots d = 6 μm and SNR = ∞. All plots use the ring‐ball structure for testing. (a) Velocity versus perfusion fraction with; (b) diffusivity versus velocity; (c) diffusivity versus perfusion fraction.

**TABLE 2:** Performance comparison of four regression models (RMSE ± standard deviation) for v, $V_{f}$, and D under noise‐free and noise conditions, showing MC‐based MLP as the superior regressor for parameter estimation.

Parameter	SNR	MC‐based MLP	MC‐based RF	IVIM‐based MLP	IVIM‐based RF
v	∞	**0.079 ± 0.024**	0.118 ± 0.035	0.249 ± 0.071	0.260 ± 0.073
v	20	**0.126 ± 0.038**	0.163 ± 0.048	0.247 ± 0.071	0.260 ± 0.073
$V_{f}$	∞	**0.052 ± 0.016**	0.072 ± 0.022	0.183 ± 0.056	0.190 ± 0.011
$V_{f}$	20	**0.097 ± 0.030**	0.119 ± 0.036	0.189 ± 0.057	0.193 ± 0.059
D	∞	**0.0002 ± 0.00006**	0.0003 ± 0.00009	0.0005 ± 0.00014	0.0006 ± 0.00018
D	20	**0.0003 ± 0.00009**	0.0004 ± 0.00011	0.0005 ± 0.00014	0.0006 ± 0.00018

*Note:* Bolded numbers are the best performance.

The biologically relevant placental capillary parameter ranges could likely contribute to the limited performance of the IVIM‐based model. Table 1 shows that D* deviates from literature‐reported feto‐placenta capillary parameters [25, 26], indicating that some values violate the IVIM assumption that D* ≫ D [11]. Thus, to evaluate capillary flows in placenta with better accuracy and stability, the MC‐based MLP was selected to apply to in vivo data. Because the models were trained on velocities within the range (0, 1] mm/s, reliable inference is confined to this interval. Velocity maps were therefore restricted to ≤ 1 mm/s. Higher‐velocity flows (Figure S4), such as those in non‐capillary vessels [39] or the intervillous space [40], due to the natural limitation of supervised machine learning, are projected to the high‐velocity region of [0.9, 1] mm/s. We also show that an MLP regressor trained only using the ring‐ball structure dataset leads to unrealistic maps with large regions of zero velocity (Figure S5), further supporting our earlier observations that both structures are required (Figure S1).

### 3.2 Parameter Prediction Using in Vivo Data

This section reports placental capillary flow velocity, perfusion fraction, and diffusion coefficient predictions using the trained Monte Carlo simulation based MLP regressor (from Section 3.1) applied to 8 in vivo scans (from Section 2.4) across gestation and compares the gestational trends across all 78 scans with IVIM parameters estimated using a least‐squares approach.

Figure 3 presents MC–based MLP regressor predictions of capillary flow velocity, perfusion fraction, and diffusion coefficient across gestation. Results of some other gestation weeks are also shown in Figure S6, and the performance of IVIM‐based MLP regressor is also shown in Figure S7. Regions of high velocity are more frequently observed during earlier gestational weeks, while lower values become more prevalent at later stages. Conversely, high‐perfusion regions are more common in later weeks, whereas low‐perfusion regions are more frequently seen during early gestation. Results for additional gestational ages are shown in Figure S6, and the performance of IVIM‐based MLP regressor is shown in Figure S7.

**FIGURE 3:**
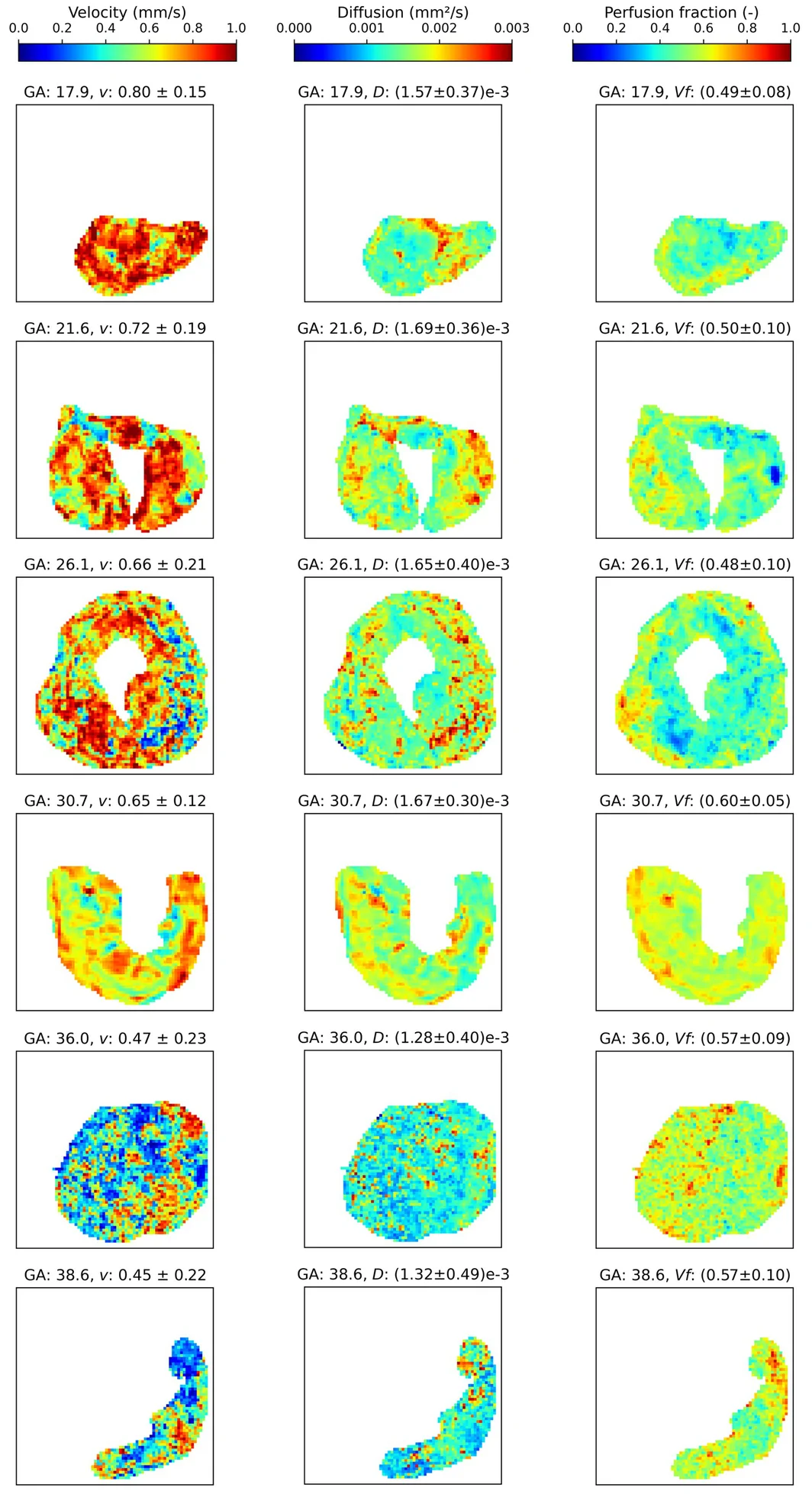
Prediction of capillary velocity (***v***), perfusion fraction ($V_{f}$) and diffusion coefficient (***D***) (mean ± standard error) using dMRI scans across gestational age (17.9–38.6 weeks), generated using an MLP regressor trained on simulated signals from both ring‐ball and randomly oriented cylinder structures.

Figure 4 summarizes the estimates of mean capillary velocity, perfusion fraction, and diffusion coefficient across all 78 scans, as obtained using the MC‐generated MLP regressor and a standard two‐step IVIM with least‐squares fitting [37] directly to the scan data. For the IVIM model, as capillary length (***L***) is unknown for in vivo data, we used a reported mean placenta capillary length (***L*** = 45 μm) [25] to estimate capillary flow velocity from the pseudo‐diffusion coefficient using the Einstein relation (D* = ***L v***/6). The MC‐generated MLP regressor predicted mean placenta capillary velocity ranging from about 0.40 to 0.90 mm/s across gestation. In contrast, the least‐squares IVIM fitting velocity estimates were an order of magnitude higher, with mean capillary velocity ranging from 0.4 to 16 mm/s. For the perfusion fraction, IVIM least squares fitting showed no significant trend across gestation. The MC‐based MLP regressor predicted a slight increasing trend, as previously [36], although this did not reach statistical significance. Both IVIM least‐squares method and MC‐based MLP regressor showed a consistent but not statistical significance decreasing trend of diffusion coefficient, which has been reported [41]. This could indicate that these parameters may remain relatively constant throughout gestation, or that any physiological changes are not detectable within our current approach.

**FIGURE 4:**
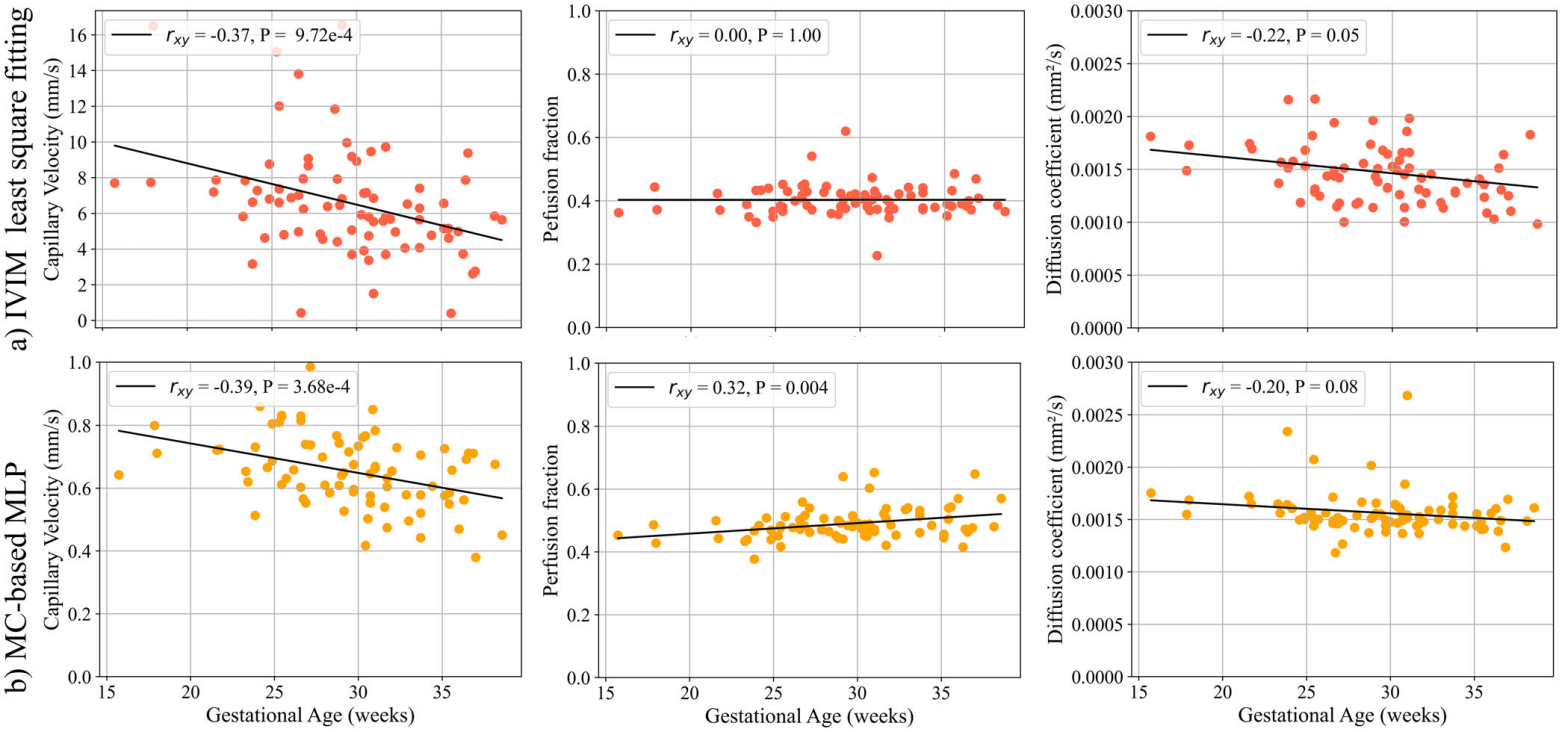
Placenta mean velocity, perfusion fraction and diffusion coefficient prediction trends across the gestational age (GA) for all participants, obtained using two approaches: (a) estimation using least squares IVIM fitting and the Einstein relation (b) predictions from the Monte Carlo simulation‐generated MLP regressor ($r_{\mathrm{xy}}$ is the slope of the line, P is the *p* value of the dataset).

## 4 Discussion and Conclusion

This study introduced an approach to estimate capillary flow velocity by combining MC simulation and supervised machine learning, enabling reliable and robust predictions including in the presence of noise. Our method utilizes biologically‐inspired simulations to predict capillary flow velocities and, notably, our estimates match previously reported measurements of mean net velocity from phase contrast angiography (≈0.6 mm/s) [8]. The consistency between these two estimates might indicate that complex, high‐spatially varying patterns within the placenta microenvironment, characterized by varying local sub‐voxel flow velocities, aggregate into a consistent mean voxel value. This numerical alignment might also indicate a form of physiological homeostasis or mass balance between fetal and maternal flow patterns. However, this comparison remains highly speculative and requires further investigation to determine if these distinct physical principles naturally converge. Compared with IVIM‐based models, which assume D* ≫ D, our results suggest that a framework with parameter regimes where D* and D are closer in magnitude can reveal perfusion‐related information that is obscured by classical IVIM models. This is likely true in the placenta, where slowly perfusing maternal blood may result in D* values approaching D. Since we cannot access ground truth microstructural and flow parameters in the complex placental environment, we cannot directly validate our estimated parameters. To mitigate this, we designed our simulation‐driven approach to capture specific physiological features of the placenta and incorporated diverse capillary geometries representative of in vivo variability. While this does not provide direct validation, it helps ensure that the estimated parameters, including capillary flow velocities, are physiologically plausible.

When applied to in vivo placental dMRI data, the proposed method demonstrated the ability to estimate spatial variations in capillary flow velocity while maintaining physiologically plausible values compared with conventional IVIM‐based approaches. Placenta velocity maps (Figure 3) show that tissue within the uterine wall generally exhibits higher blood flow. A decreasing velocity trend was observed both in velocity maps (Figure 3) and the mean velocity trend plot (Figure 4) during gestation, which could potentially reflect increased small vessel density as gestation progresses [42]. More broadly, compared with traditional fitting approaches that can yield unrealistically high perfusion estimates, the literature‐consistent velocity range produced by the proposed framework suggests improved physiological plausibility. These insights may improve understanding of the development and maturation of placental vasculature and support non‐invasive investigation of placental blood flow dynamics.

The primary aim of this work is to identify a simplified model capable of isolating at least one robust physiological parameter (i.e., capillary flow velocity) from dMRI to quantify placenta functions. Therefore, models were trained on capillary velocities within the range (0, 1] mm/s. Since predictive performance in supervised learning approaches is typically constrained by the range represented in the training data, when applied to higher velocities, estimation accuracy decreases, reflecting limited extrapolation beyond the training distribution. Consequently, higher‐velocity flows, such as those occurring in larger vessels [39] or the intervillous space (reported as ranging from 1 to 350 mm/s [40]) will be projected into the high capillary velocity region of [0.9, 1] mm/s. This likely results in an overestimation of mean capillary velocity, as some velocities in this region may reflect either rapidly flowing fetal blood in large vessels or maternal blood flow. While this work focuses on the capillary side, maternal blood flow within the intervillous space represents a distinct physiological component. Characterizing these complex, non‐vessel interactions remains a key future objective. The framework will also be extended to incorporate additional tissue components, such as cells, and microstructural features. For example, as our ring‐ball and randomly orientated cylinder structures represent only the terminal capillary networks of the villous tree, future work will aim to incorporate larger‐scale villous tree geometries into the flow simulations, such as those described in our team's previous work [39]. Thus, future work will focus on integrating established oxygen and nutrient transport modeling frameworks for placental capillaries [43] into our existing velocity simulations. This integration will enable us to directly test how variations in relative maternal and fetal blood velocities affect physiological exchange efficiency.

Our simulations utilize constant segment length and diameter based on term‐placenta ranges, as robust non‐term data remains limited. Our velocity predictions are averages over the voxel‐size (˜3 mm^3^) which is significantly larger than the scale of capillary structures (˜200 μm). Future gestation‐specific structures will incorporate regional and gestational variations in capillary structures to enhance biological specificity [44].

Figure 4a shows that least‐squares IVIM fitting yielded velocity estimates that were an order of magnitude higher than the simulation‐based approach, with mean capillary velocities ranging from 0.4 to 16 mm/s. These velocity values are significantly higher than those dictated by physical boundary laws in the placental microvasculature [26], indicating that the traditional assumption that D* ≫ ***D*** may break down in this organ due to the complex physiology of placental perfusion. This might also suggest that historical literature reporting high in vivo placental D* values may be capturing macro‐perfusion in larger vessels or the intervillous space, rather than isolated capillary networks. Our study uses only relatively simple synthetic capillary structures while including more different structures potentially helps the MLP regressors to extract relevant features. In the future, we will develop more complex and representative in silico capillary structures [17, 18, 19] to enhance the diversity of our simulated dataset and further refine our approach. Additionally, we will conduct a more rigorous evaluation using MC simulations of flow in highly complex capillary geometries that differ from those seen during training. This will allow us to assess how well our method generalizes to unseen capillary structures—an essential consideration, as the true in vivo capillary architecture of the placenta remains unknown. We could also validate our approach using micro‐CT images of ex vivo placentas [41], though this comes with the limitation that placental structure changes significantly after delivery.

In conclusion, this method gives reasonable in vivo parameter estimates that support expected spatial and gestational trends in placental MRI and captures meaningful changes across gestation which are otherwise difficult to capture using IVIM‐based approaches. With further validation and enhancement, this approach is adaptable and applicable to various organs where blood flow velocity plays a significant role, offering a non‐invasive means to study blood flow dynamics in both physiological and pathological conditions, with potential clinical applications in monitoring placental health and detecting perfusion abnormalities.

## Funding

This work was supported by King's College London Medical Engineering Centre of Research Excellence, WT203148/Z/16/Z; DFG Heisenberg, 502024488; UKRI Future Leaders Fellowship Renewal, MR/T018119/1, MR/T020296/2; Medical Research Council (MRC), MR/T046473/1; Cancer Research UK, C44767/A29458; Wellcome Leap In Utero award; Engineering and Physical Sciences Research Council (EPSRC), EP/V034537/1.

## Conflicts of Interest

The authors declare no conflicts of interest. The authors confirm they have read and understood the journal's conflict of interest policy and that this disclosure is complete and accurate to the best of their knowledge.

## Supporting information


**Figure S1:** Plot of the first two principal components of PCA applied to simulated dMRI signals from ring‐ball and randomly orientated cylinder structures.
**Figure S2:** Prediction bias maps based on Monte Carlo simulation‐generated and IVIM‐generated RF and MLP regressors under *d* = 6 μm with noise (SNR = 20), shown (a) velocity versus perfusion fraction; (b) diffusivity versus velocity; (c) diffusivity versus perfusion fraction (using the ring‐ball structure for testing).
**Figure S3:** Prediction bias maps based on Monte Carlo simulation‐generated and IVIM‐generated RF and MLP regressors under *d* = 6 μm with noise (SNR = 20), shown (a) velocity versus perfusion fraction; (b) diffusivity versus velocity; (c) diffusivity versus perfusion fraction (using the randomly oriented cylinder structure for testing).
**Figure S4:** Prediction bias maps of high blood velocity (2–10 mm/s) versus perfusion fraction using Monte Carlo simulation‐generated (the ring‐ball structure) and IVIM‐generated RF and MLP regressors under *d* = 6 μm without noise (SNR = ∞), using the ring‐ball structure test dataset.
**Figure S5:** Velocity predictions from the MLP regressor with different gestational age, given ring‐ball structure signals only.
**Figure S6:** Prediction of capillary velocity (***v***), perfusion fraction ($V_{f}$) and diffusion coefficient (***D***) (mean ± standard deviation) using dMRI scans at 24.9 and 31.7 gestation weeks, generated using an MLP regressor trained on simulated signals from both ring‐ball and randomly oriented cylinder structures.
**Figure S7:** Prediction of capillary velocity (***v***), perfusion fraction ($V_{f}$) and diffusion coefficient (***D***) (mean ± standard deviation) using dMRI scans across gestational age (17.9–38.6 weeks), generated using an MLP regressor trained on IVIM‐generated signals.

## Data Availability

The data that support the findings of this study are available on request from the corresponding author. The data are not publicly available due to privacy or ethical restrictions.
