# Supplementary material for: Placental Blood‐Flow Velocity Quantification From Diffusion MRI
Source: Magn Reson Med. 2026 Aug 10;96(6):2967–77. doi: 10.1002/mrm.70551 (PMC13619870; doi:10.1002/mrm.70551)
Supplement: Supplementary file 1 [file MRM-96-2967-s001.docx]

Supplementary Material

As illustrated in Figure S1, principal component analysis (PCA) was applied to the simulated signals from ring-ball and randomly orientated geometries by projecting them into a shared 2D feature space to examine their representation distribution. The first two components account for 77.2% and 12.8% of the total variance, respectively, capturing most signal variability and clearly showing separation between the two capillary structures. Identical velocities map to distinct locations in PCA space, demonstrating sensitivity of the dMRI signal to the two different microvascular geometries. Consequently, neither structure alone supports unambiguous velocity estimation. Joint consideration of multiple structures provides complementary information that can reduce ambiguities.

Figure S1. Plot of the first two principal components of PCA applied to simulated dMRI signals from ring-ball and randomly orientated cylinder structures.

Figure S2. Prediction bias maps based on Monte Carlo simulation-generated and IVIM-generated RF and MLP regressors under $\boldsymbol{d}$ = 6 µm with noise (SNR = 20), shown (a) velocity vs perfusion fraction; (b) diffusivity vs velocity; (c) diffusivity vs perfusion fraction (using the ring-ball structure for testing).

 Figure S3. Prediction bias maps based on Monte Carlo simulation-generated and IVIM-generated RF and MLP regressors under $\boldsymbol{d}$ = 6 µm with noise (SNR =$20$), shown (a) velocity vs perfusion fraction; (b) diffusivity vs velocity; (c) diffusivity vs perfusion fraction (using the randomly oriented cylinder structure for testing).

Figure S4. Prediction bias maps of high blood velocity (2-10 mm/s) vs perfusion fraction using Monte Carlo simulation-generated (the ring-ball structure) and IVIM-generated RF and MLP regressors under $\boldsymbol{d}$ = 6 µm without noise (SNR =$\infty$), using the ring-ball structure test dataset.

As illustrated in Figure S5, given ring-ball signals only to train the MLP regressor, the velocity predictions across the gestation show limited velocity features but large regions have capillary velocity close to zero.

Figure S5. Velocity predictions from the MLP regressor with different gestational age, given ring-ball structure signals only.

Figure S6. Prediction of capillary velocity (***v***), perfusion fraction ($\boldsymbol{V}_{\boldsymbol{f}}$) and diffusion coefficient (***D***) (mean ± standard deviation) using dMRI scans at 24.9 and 31.7 gestation weeks, generated using an MLP regressor trained on simulated signals from both ring-ball and randomly oriented cylinder structures.

Figure S7. Prediction of capillary velocity (***v***), perfusion fraction ($\boldsymbol{V}_{\boldsymbol{f}}$) and diffusion coefficient (***D***) (mean ± standard deviation) using dMRI scans across gestational age (17.9–38.6 weeks), generated using an MLP regressor trained on IVIM-generated signals.
